# Crystal structure and Hirshfeld surface analysis of 2,4-di­amino-6-phenyl-1,3,5-triazin-1-ium 4-methyl­benzene­sulfonate

**DOI:** 10.1107/S2056989018010368

**Published:** 2018-07-27

**Authors:** Ramalingam Sangeetha, Kasthuri Balasubramani, Kaliyaperumal Thanigaimani, Savaridasson Jose Kavitha

**Affiliations:** a Department of Chemistry, Governemnt Arts College (Autonomous), Karur 639 005, Tamil Nadu, India; b Department of Chemistry, Government Arts College, Thiruchirappalli 620 022, Tamil Nadu, India; c Department of Chemistry, Mother Teresa Womens University, Kodaikanal 624 102, Tamil Nadu, India

**Keywords:** crystal structure, triazinium cation, 4-methyl­benzene­sulfonate anion, *DDAA* array, Hirshfeld surface analysis

## Abstract

In the title mol­ecular salt, C_9_H_10_N_5_^+^·C_7_H_7_O_3_S^−^, the asymmetric unit consists of a 2,4-di­amino-6-phenyl-1,3,5-triazin-1-ium cation and a 4-methyl­benzene­sulfonate anion. The cation is protonated at the N atom lying between the amine and phenyl substituents. The protonated N and amino-group N atoms are involved in hydrogen bonding with the sulfonate O atoms through a pair of inter­molecular N—H⋯O hydrogen bonds, giving rise to a hydrogen-bonded cyclic motif with *R*_2_^2^(8) graph-set notation. The inversion-related mol­ecules are further linked by four N—H⋯O inter­molecular inter­actions to produce a complementary *DDAA* (*D* = donor, *A* = acceptor) hydrogen-bonded array, forming *R*_2_^2^(8), *R*_4_^2^(8) and *R*_2_^2^(8) ring motifs. The centrosymmetrically paired cations form *R*_2_^2^(8) ring motifs through base-pairing *via* N—H⋯N hydrogen bonds. In addition, another *R*_3_^3^(10) motif is formed between centrosymetrically paired cations and a sulfonate anion *via* N—H⋯O hydrogen bonds. The crystal structure also features weak S=O⋯π and π–π inter­actions. Hirshfeld surface and fingerprint plots were employed in order to further study the inter­molecular inter­actions.

## Abstract 2

The asymmetric unit consists of a 2,4-di­amino-6-phenyl-1,3,5-triazin-1-ium cation and a 4-methyl­benzoate anion. The protonated nitro­gen and amino group nitro­gen atoms are involved in hydrogen bonding with the sulfonate oxygen atoms through a pair of inter­molecular N—H⋯O hydrogen bonds. The inversion-related mol­ecules are further linked by four N—H⋯O inter­molecular inter­ations to produce a complementary *DDAA* hydrogen-bonded array. Hirshfeld surface analysis was employed to further examine the inter­molecular inter­actions.

## Chemical context   

Triazine derivatives have been found to possess a wide variety of biological activities such as anti­cancer (El-Gendy *et al.*, 2001 ▸; Abdel-Rahman *et al.*, 1999 ▸), anti­tumour (Menicagli *et al.*, 2004 ▸) and anti-inflammatory (El-Massry *et al.*, 1999 ▸) activities. In addition, many *s*-triazine derivatives have been found to exhibit anti­bacterial (Jyoti *et al.*, 2003 ▸) and herbicidal activity. The 1,3,5-triazine moieties are of particular inter­est because of their potentially large non-linear optical response (Marchewka *et al.*, 2003 ▸). Triazine derivatives of melamine and benzoguanamine are used to manufacture resins (Ricciotti *et al.*, 2013 ▸). They are used as preservatives in oil-field applications and as disinfectants, industrial deodorants and as a biocide in water treatments. Triazine derivatives have been used appreciably as a valuable constructing unit of subtle architectures consisting of organic and inorganic hybrid frameworks (Ma­thias *et al.*, 1994 ▸; Zerkowski *et al.*, 1994 ▸; MacDonald & Whitesides, 1994 ▸; Guru Row *et al.*, 1999 ▸; Krische & Lehn, 2000 ▸; Sherrington & Taskinen, 2001 ▸). Herein the crystal structure of 2,4-di­amino-6-phenyl-1,3,5-triazine-1-ium-4-methyl­benzene sulfonate is described. Hirshfeld surface analysis and two-dimensional fingerprint plots were employed to qu­antify the percentage contributions of the inter­molecular inter­actions present in the mol­ecule.
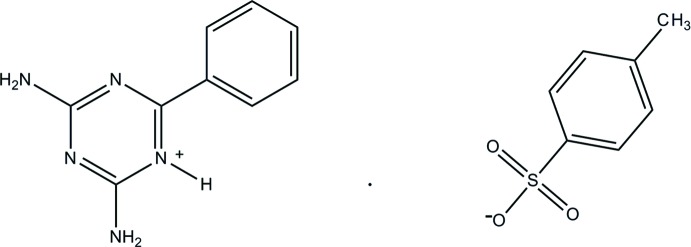


## Structural commentary   

**Figure 1:**
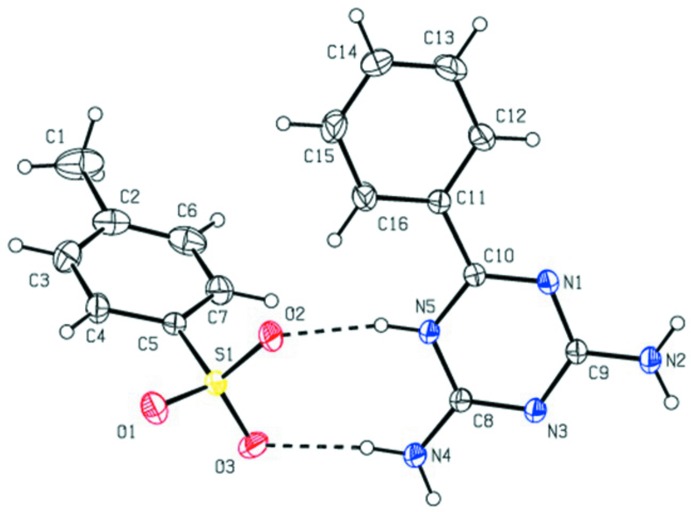
The mol­ecular structure of the title compound with displacement ellipsoids drawn at the 40% probability level. N—H⋯O hydrogen bonds (dashed lines) form an 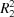
(8) ring motif between the 2,4-di­amino-6-phenyl-1,3,5-triazin-1-ium cation and 4-methyl­benzene­sulfonate anion.

The mol­ecular structure with its atomic numbering scheme is shown in Fig. 1 ▸. The asymmetric unit comprises a 2,4-di­amino-6-phenyl-1,3,5-triazin-1-ium cation and a 4-methyl­benzene sulfonate anion. The cation is protonated at atom N5, which lies between the amine and phenyl substituents: this proton­ation is reflected by an increase in the bond angle at N5 [C8—N5—C10 = 119.43 (15)°] compared to the unprotonated atom N3 [C8—N3—C9 = 115.88 (15)°] and the corresponding angle of 113.7 (4)° in neutral 2,4-di­amino-6-phenyl-1,3,5-triazine (Díaz-Ortiz *et al.*, 2004 ▸). Otherwise, bond lengths and angles are in normal ranges (Allen *et al.*, 1987 ▸).

## Supra­molecular features   

**Figure 2:**
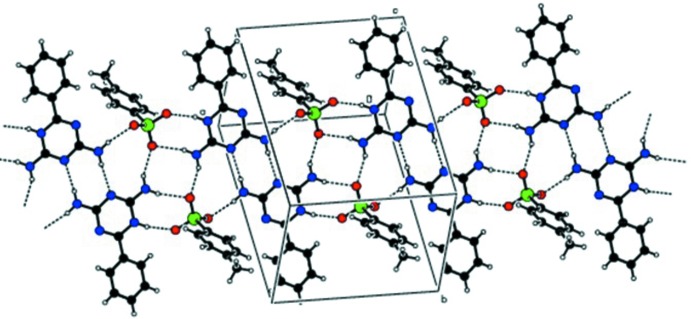
Crystal packing of the title compound viewed along the *b* axis. Dashed lines indicate N—H⋯O and N—H⋯N hydrogen bonds, which form a complementary DDAA hydrogen bonded-array with 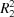
(8), 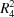
(8), 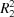
(8) and 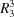
(10) graph-set motifs, generating a one-dimensional hydrogen-bonded supra­molcular structure. (Red = oxygen, green = sulfur).

In the crystal, the protonated nitro­gen (N5) and amino group nitro­gen (N4) atoms are involved in hydrogen bonding with the 4-methyl­benzene sulfonate oxygen atoms O2 and O3 through a pair of inter­molecular N—H⋯O hydrogen bonds, giving rise to a hydrogen-bonded 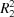
(8) cyclic graph-set motif (Fig. 1 ▸, Table 1 ▸). Here the sulfonate oxygen atoms mimic the role of carboxyl­ate oxygen atoms. The inversion-related mol­ecules are further linked by four N—H⋯O hydrogen bonds, forming an another 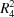
(8) ring motif to produce a *DDAA* array of quadruple hydrogen bonds. This type of conjoined hydrogen-bonded ring motifs can be represented as 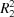
(8), 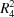
(8) and 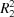
(8), repectively (Fig. 2 ▸). The inversion-related triazinium bases are paired by two N—H⋯N hydrogen bonds, generating an 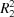
(8) graph-set motif. In addition, another 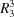
(10) ring motif is formed between centrosymetrically paired cations and a sulfonate anion *via* N—H⋯O hydrogen bonds. One of the sulfonate oxygen atoms acts as an acceptor of bifurcated hydrogen bonds. Overall, these hydrogen bonds generate chains along (100).

**Figure 3:**
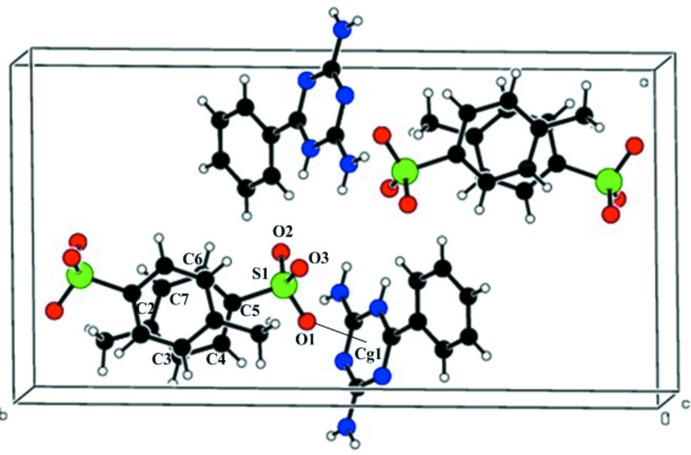
A packing view along the *c* axis showing the weak inter­molecular S1= O1⋯*Cg*1 (dashed line) and π–π inter­actions.

A weak inter­molecular π–ring inter­action between atom O1 of the anion and the π-system of the triazinium ring is observed in a slipped-parallel mode [S1—O1⋯*C*g1; *Y*—*X*, π = 46.33°], (Fig. 3 ▸, Table 1 ▸). A similar inter­action was observed in 1,3-dimeth­oxy-2-methyl­imidazolium bis­(tri­fluoro­methane­sulfon­yl)imide (Partl *et al.*,2016 ▸). π–π inter­actions are also observed between the anionic rings, with a centroid-to-centroid distance of 3.9192 (13) Å.

## Hirshfeld surface analysis   

**Figure 4:**
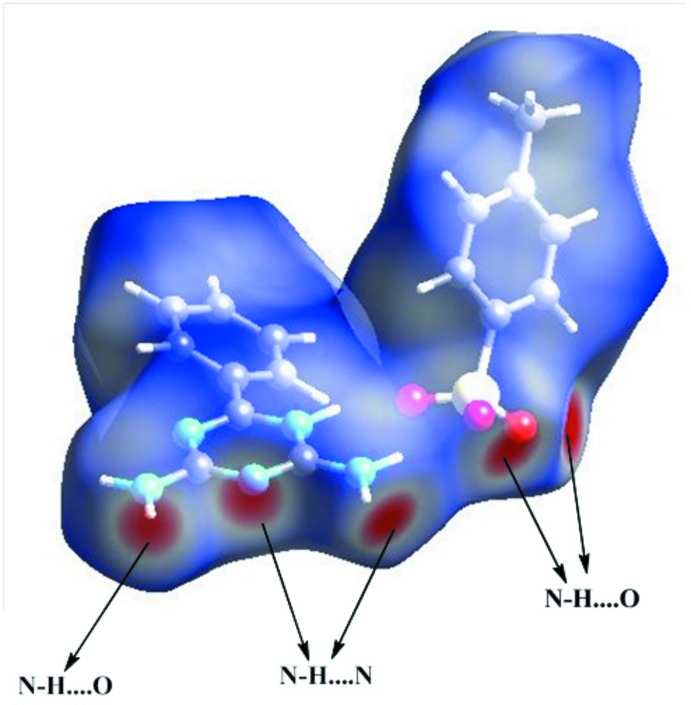
A view of the three-dimensional Hirshfeld surface of the title compound.

**Figure 5:**
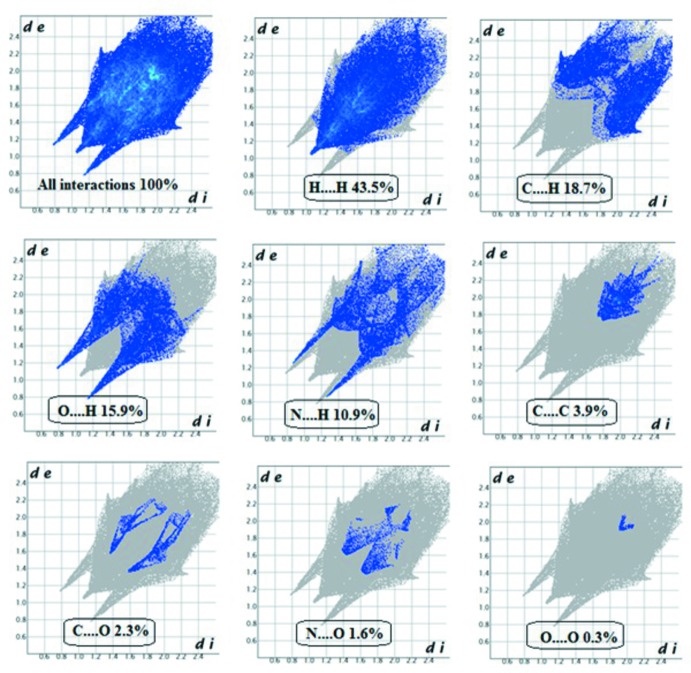
Two-dimensional fingerprint plots for the title compound.

Hirshfeld surface analysis (Spackman & Jayatilaka, 2009 ▸) and two-dimensional fingerprint plots are useful tools for describing the surface characteristics of the crystal structure and were generated using *CrystalExplorer3.0* (Wolff *et al.*, 2012 ▸). The normalized contact distance (*d*_norm_) is based on the distances from the nearest atom inside (*d*_i_) and outside (*d*_e_) the surface. The three-dimensional *d*_norm_ surface of the title compound is shown in Fig. 4 ▸. The red points represent closer contacts and negative *d*_norm_ values on the surface corres­ponding to N—H⋯O and N—H⋯N inter­actions. Two-dimensional fingerprint plots are shown in Fig. 5 ▸. The H⋯H inter­actions (43.5%) and C⋯H (18.7%) inter­actions make the highest contributions with the O⋯H (15.9%) N⋯H (10.9%), C⋯C (3.9%), C⋯O (2.3%), N⋯O (1.6%) and O⋯O (0.3%) contacts also making significant contributions to the Hirshfeld surface.

## Database survey   

A search of the Cambridge Structural Database (Version 5.37, update February 2016 Groom *et al.*, 2016 ▸) for 2,4-di­amino-6-phenyl-1,3,5-triazine yielded five crystal structures of proton-transfer salts with carb­oxy­lic acids: HEVQAB (with oxalic acid; Aghabozorg *et al.*, 2006 ▸), HEWFOG (with picric acid; Goel *et al.*, 2013 ▸), TEZNAP (with phthalic acid; Delori *et al.*, 2013 ▸), WEPBUP (with hydrogen chloride; Sheshmani *et al.*, 2006 ▸), and YOCZOH (with 2,3,5,6-tetra­fluoro­terephthalic acid; Wang *et al.*, 2014 ▸).

## Synthesis and crystallization   

The title compound was prepared by mixing a hot methano­lic solution (20 ml) of 2,4-di­amino-6-phenyl-1,3,5-triazine (0.187 g) and a hot methano­lic solution (10 ml) of 4-methyl­benzene sulfonic acid (0.172 g) in 1:1 molar ratio. The reaction mixture was warmed over a water bath for a few minutes. The resultant solution was then allowed to cool slowly at room temperature. After a few days, colourless block-shaped crystals were separated out.

## Refinement   

Crystal data, data collection and structure refinement details are summarized in Table 2 ▸. The C- and N- bound H atoms were placed in calculated positions and were included in the refinement in the riding-model approximation: C—H = 0.93 Å and N—H = 0.86 Å with *U*_iso_(H) set to 1.2–1.5*U*_eq_(C) or 1.3*U*_eq_(N).

## Supplementary Material

Crystal structure: contains datablock(s) global, I. DOI: 10.1107/S2056989018010368/jj2200sup1.cif

Supporting information file. DOI: 10.1107/S2056989018010368/jj2200Isup2.cml

CCDC reference: 1820866

Additional supporting information:  crystallographic information; 3D view; checkCIF report

## Figures and Tables

**Table 1:** Hydrogen-bond geometry (Å, °) *Cg*1 and *Cg*3 are the centroids of the N1/C9/N3/C8/N5/C10 and C2–C5/C6/C7 rings, respectively.

*D*—H⋯*A*	*D*—H	H⋯*A*	*D*⋯*A*	*D*—H⋯*A*
N4—H2*N*4⋯O3^i^	0.86	2.10	2.877 (2)	150
N4—H1*N*4⋯O3	0.86	2.13	2.950 (2)	160
N2—H2*N*2⋯N3^ii^	0.86	2.25	3.089 (2)	164
N2—H1*N*2⋯O1^iii^	0.86	2.05	2.895 (2)	169
N5—H1*N*5⋯O2	0.86	1.95	2.789 (2)	165
C16—H16⋯O2	0.93	2.40	3.210 (3)	146
S1—O1⋯*Cg*1^iv^		2.93 (1)	4.1695 (8)	142 (1)
*Cg*3—*Cg*3			3.9192 (13)	

Symmetry codes: (i) 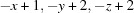; (ii) 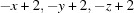; (iii) 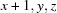; (iv) 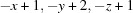; (v) 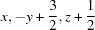.

**Table 2:** Experimental details

Crystal data	
Chemical formula	C_9_H_10_N_5_^+^·C_7_H_7_O_3_S^−^
*M*_r_	359.41
Crystal system, space group	Monoclinic, *P*2_1_/*c*
Temperature (K)	296
*a*, *b*, *c* (Å)	11.0060 (6), 20.7269 (11), 7.6213 (4)
β (°)	97.468 (2)
*V* (Å^3^)	1723.83 (16)
*Z*	4
Radiation type	Mo *K*α
μ (mm^−1^)	0.21
Crystal size (mm)	0.35 × 0.35 × 0.30

Data collection	
Diffractometer	Bruker Kappa APEXII CCD
Absorption correction	Multi-scan (*SADABS*; Bruker, 2004 ▸)
*T*_min_, *T*_max_	0.929, 0.939
No. of measured, independent and observed [*I* > 2σ(*I*)] reflections	20842, 4273, 3325
*R*_int_	0.033
(sin θ/λ)_max_ (Å^−1^)	0.667

Refinement	
*R*[*F* ^2^ > 2σ(*F* ^2^)], *wR*(*F* ^2^), *S*	0.049, 0.151, 1.01
No. of reflections	4277
No. of parameters	227
H-atom treatment	H-atom parameters constrained
Δρ_max_, Δρ_min_ (e Å^−3^)	0.49, −0.43

Computer programs: *APEX2*, *SAINT* and *XPREP* (Bruker, 2004 ▸), *SHELXS97* and *SHELXL97* (Sheldrick, 2008 ▸) and *PLATON* (Spek, 2009 ▸).

## supplementary crystallographic information

### Crystal data

**Table:**

C_9_H_10_N_5_^+^·C_7_H_7_O_3_S^−^	*F*(000) = 752
*M**_r_* = 359.41	*D*_x_ = 1.385 Mg m^−^^3^*D*_m_ = 1.381 Mg m^−^^3^*D*_m_ measured by Not Measured
Monoclinic, *P*2_1_/*c*	Mo *K*α radiation, λ = 0.71073 Å
Hall symbol: -P 2ybc	Cell parameters from 6410 reflections
*a* = 11.0060 (6) Å	θ = 5.7–56.4°
*b* = 20.7269 (11) Å	µ = 0.21 mm^−^^1^
*c* = 7.6213 (4) Å	*T* = 296 K
β = 97.468 (2)°	Block, colourless
*V* = 1723.83 (16) Å^3^	0.35 × 0.35 × 0.30 mm
*Z* = 4	

### Data collection

**Table:**

Bruker Kappa APEXII CCD diffractometer	4273 independent reflections
Radiation source: fine-focus sealed tube	3325 reflections with *I* > 2σ(*I*)
Graphite monochromator	*R*_int_ = 0.033
Detector resolution: 18.4 pixels mm^-1^	θ_max_ = 28.3°, θ_min_ = 2.7°
ω and φ scan	*h* = −14→14
Absorption correction: multi-scan (SADABS; Bruker, 2004)	*k* = −27→24
*T*_min_ = 0.929, *T*_max_ = 0.939	*l* = −9→10
20842 measured reflections	

### Refinement

**Table:**

Refinement on *F*^2^	Primary atom site location: structure-invariant direct methods
Least-squares matrix: full	Secondary atom site location: difference Fourier map
*R*[*F*^2^ > 2σ(*F*^2^)] = 0.049	Hydrogen site location: inferred from neighbouring sites
*wR*(*F*^2^) = 0.151	H-atom parameters constrained
*S* = 1.01	*w* = 1/[σ^2^(*F*_o_^2^) + (0.0845*P*)^2^ + 0.7378*P*] where *P* = (*F*_o_^2^ + 2*F*_c_^2^)/3
4277 reflections	(Δ/σ)_max_ = 0.004
227 parameters	Δρ_max_ = 0.49 e Å^−^^3^
0 restraints	Δρ_min_ = −0.43 e Å^−^^3^

### Special details

**Table:**

Geometry. Bond distances, angles etc. have been calculated using the rounded fractional coordinates. All su's are estimated from the variances of the (full) variance-covariance matrix. The cell esds are taken into account in the estimation of distances, angles and torsion angles
Refinement. Refinement on F^2^ for ALL reflections except those flagged by the user for potential systematic errors. Weighted R-factors wR and all goodnesses of fit S are based on F^2^, conventional R-factors R are based on F, with F set to zero for negative F^2^. The observed criterion of F^2^ > 2sigma(F^2^) is used only for calculating -R-factor-obs etc. and is not relevant to the choice of reflections for refinement. R-factors based on F^2^ are statistically about twice as large as those based on F, and R-factors based on ALL data will be even larger.

### Fractional atomic coordinates and isotropic or equivalent isotropic displacement parameters (Å^2^)

**Table:**

	*x*	*y*	*z*	*U*_iso_*/*U*_eq_	
S1	0.34811 (4)	0.91624 (2)	0.65382 (7)	0.0341 (2)	
N1	0.90645 (14)	0.92499 (8)	0.5823 (2)	0.0331 (5)	
N2	1.04787 (14)	0.97433 (9)	0.7826 (2)	0.0413 (5)	
N3	0.85035 (14)	0.97568 (8)	0.8454 (2)	0.0328 (5)	
N4	0.64605 (15)	0.97966 (9)	0.8776 (2)	0.0449 (6)	
N5	0.70324 (13)	0.92814 (7)	0.6349 (2)	0.0306 (4)	
O1	0.24577 (13)	0.95410 (7)	0.5729 (2)	0.0463 (5)	
O2	0.45015 (13)	0.91551 (8)	0.5499 (2)	0.0506 (5)	
O3	0.38962 (15)	0.93469 (8)	0.8365 (2)	0.0519 (5)	
C8	0.73449 (16)	0.96182 (9)	0.7882 (2)	0.0313 (5)	
C9	0.93250 (16)	0.95848 (9)	0.7378 (2)	0.0307 (5)	
C10	0.79160 (16)	0.91143 (8)	0.5351 (2)	0.0295 (5)	
C11	0.75537 (17)	0.87572 (10)	0.3686 (3)	0.0347 (5)	
C12	0.8406 (2)	0.83807 (18)	0.3023 (4)	0.0817 (13)	
C13	0.8094 (3)	0.8038 (2)	0.1478 (5)	0.1184 (18)	
C14	0.6952 (3)	0.80698 (17)	0.0582 (4)	0.0729 (10)	
C15	0.6098 (2)	0.84433 (17)	0.1229 (3)	0.0669 (9)	
C16	0.6392 (2)	0.87874 (14)	0.2786 (3)	0.0541 (8)	
C1	0.1803 (4)	0.63794 (13)	0.6294 (4)	0.0804 (13)	
C2	0.2180 (3)	0.70767 (11)	0.6442 (3)	0.0510 (8)	
C3	0.1408 (2)	0.75664 (11)	0.5771 (3)	0.0518 (8)	
C4	0.17777 (18)	0.82075 (10)	0.5852 (3)	0.0407 (6)	
C5	0.29530 (17)	0.83591 (9)	0.6579 (2)	0.0318 (5)	
C6	0.3336 (3)	0.72421 (12)	0.7230 (3)	0.0560 (8)	
C7	0.3737 (2)	0.78743 (11)	0.7282 (3)	0.0478 (7)	
H2N4	0.66290	1.00070	0.97500	0.0540*	
H1N4	0.57120	0.97030	0.83880	0.0540*	
H2N2	1.06960	0.99530	0.87890	0.0500*	
H1N2	1.10170	0.96380	0.71550	0.0500*	
H1N5	0.62810	0.91760	0.60200	0.0370*	
H12	0.91980	0.83550	0.36160	0.0980*	
H13	0.86800	0.77810	0.10440	0.1420*	
H14	0.67540	0.78390	−0.04630	0.0870*	
H15	0.53090	0.84680	0.06220	0.0800*	
H16	0.58000	0.90400	0.32220	0.0650*	
H1A	0.21910	0.61750	0.53820	0.1200*	
H1B	0.09300	0.63520	0.60040	0.1200*	
H1C	0.20480	0.61660	0.74020	0.1200*	
H3	0.06200	0.74650	0.52500	0.0620*	
H4	0.12350	0.85320	0.54190	0.0490*	
H6	0.38570	0.69210	0.77390	0.0670*	
H7	0.45300	0.79740	0.77850	0.0570*	

### Atomic displacement parameters (Å^2^)

**Table:**

	*U*^11^	*U*^22^	*U*^33^	*U*^12^	*U*^13^	*U*^23^
S1	0.0234 (2)	0.0389 (3)	0.0409 (3)	−0.0040 (2)	0.0079 (2)	−0.0090 (2)
N1	0.0236 (7)	0.0420 (9)	0.0334 (8)	0.0026 (6)	0.0021 (6)	−0.0055 (6)
N2	0.0245 (8)	0.0586 (11)	0.0406 (9)	−0.0045 (7)	0.0039 (7)	−0.0133 (8)
N3	0.0253 (7)	0.0397 (9)	0.0335 (8)	−0.0046 (6)	0.0041 (6)	−0.0066 (6)
N4	0.0271 (8)	0.0616 (12)	0.0476 (10)	−0.0097 (7)	0.0107 (7)	−0.0249 (8)
N5	0.0222 (7)	0.0370 (8)	0.0325 (8)	−0.0047 (6)	0.0035 (6)	−0.0067 (6)
O1	0.0344 (8)	0.0381 (8)	0.0674 (10)	0.0022 (6)	0.0108 (7)	0.0063 (7)
O2	0.0286 (7)	0.0702 (11)	0.0558 (10)	−0.0100 (7)	0.0157 (7)	−0.0144 (8)
O3	0.0491 (9)	0.0605 (10)	0.0462 (9)	−0.0117 (8)	0.0070 (7)	−0.0225 (8)
C8	0.0267 (9)	0.0337 (9)	0.0341 (9)	−0.0044 (7)	0.0062 (7)	−0.0047 (7)
C9	0.0248 (8)	0.0346 (9)	0.0321 (9)	0.0003 (7)	0.0013 (7)	−0.0009 (7)
C10	0.0254 (8)	0.0310 (8)	0.0316 (9)	0.0018 (6)	0.0018 (7)	−0.0023 (7)
C11	0.0289 (9)	0.0420 (10)	0.0331 (9)	0.0006 (8)	0.0032 (7)	−0.0071 (8)
C12	0.0373 (13)	0.125 (3)	0.078 (2)	0.0236 (15)	−0.0105 (13)	−0.062 (2)
C13	0.0559 (18)	0.187 (4)	0.107 (3)	0.037 (2)	−0.0091 (17)	−0.106 (3)
C14	0.0574 (16)	0.104 (2)	0.0551 (16)	0.0007 (15)	−0.0010 (12)	−0.0444 (16)
C15	0.0394 (13)	0.115 (2)	0.0445 (13)	−0.0081 (14)	−0.0015 (10)	−0.0290 (15)
C16	0.0315 (11)	0.0896 (19)	0.0411 (12)	0.0025 (11)	0.0040 (9)	−0.0221 (12)
C1	0.127 (3)	0.0380 (13)	0.082 (2)	−0.0115 (15)	0.036 (2)	−0.0056 (13)
C2	0.0741 (17)	0.0371 (11)	0.0458 (12)	−0.0021 (11)	0.0234 (12)	−0.0037 (9)
C3	0.0452 (12)	0.0456 (12)	0.0647 (15)	−0.0135 (10)	0.0078 (11)	−0.0042 (11)
C4	0.0290 (9)	0.0396 (11)	0.0525 (12)	−0.0024 (8)	0.0014 (8)	0.0017 (9)
C5	0.0291 (9)	0.0363 (9)	0.0302 (9)	0.0009 (7)	0.0046 (7)	−0.0066 (7)
C6	0.0739 (17)	0.0424 (12)	0.0526 (14)	0.0203 (12)	0.0120 (12)	0.0052 (10)
C7	0.0408 (12)	0.0542 (13)	0.0454 (12)	0.0129 (10)	−0.0057 (9)	−0.0053 (10)

### Geometric parameters (Å, º)

**Table:**

S1—O1	1.4437 (15)	C14—C15	1.359 (4)
S1—O2	1.4560 (15)	C15—C16	1.386 (4)
S1—O3	1.4588 (16)	C12—H12	0.9300
S1—C5	1.7651 (19)	C13—H13	0.9300
N1—C10	1.299 (2)	C14—H14	0.9300
N1—C9	1.371 (2)	C15—H15	0.9300
N2—C9	1.313 (2)	C16—H16	0.9300
N3—C8	1.324 (2)	C1—C2	1.504 (4)
N3—C9	1.345 (2)	C2—C3	1.379 (3)
N4—C8	1.312 (2)	C2—C6	1.378 (4)
N5—C10	1.355 (2)	C3—C4	1.389 (3)
N5—C8	1.366 (2)	C4—C5	1.376 (3)
N2—H2N2	0.8600	C5—C7	1.386 (3)
N2—H1N2	0.8600	C6—C7	1.382 (3)
N4—H2N4	0.8600	C1—H1A	0.9600
N4—H1N4	0.8600	C1—H1B	0.9600
N5—H1N5	0.8600	C1—H1C	0.9600
C10—C11	1.478 (3)	C3—H3	0.9300
C11—C12	1.367 (4)	C4—H4	0.9300
C11—C16	1.372 (3)	C6—H6	0.9300
C12—C13	1.380 (5)	C7—H7	0.9300
C13—C14	1.352 (5)		
			
O1—S1—O2	112.79 (9)	C11—C12—H12	120.00
O1—S1—O3	113.29 (9)	C13—C12—H12	120.00
O1—S1—C5	106.25 (9)	C12—C13—H13	119.00
O2—S1—O3	110.72 (9)	C14—C13—H13	119.00
O2—S1—C5	106.19 (9)	C15—C14—H14	120.00
O3—S1—C5	107.07 (9)	C13—C14—H14	120.00
C9—N1—C10	115.81 (15)	C14—C15—H15	120.00
C8—N3—C9	115.88 (15)	C16—C15—H15	120.00
C8—N5—C10	119.43 (15)	C15—C16—H16	120.00
C9—N2—H1N2	120.00	C11—C16—H16	120.00
C9—N2—H2N2	120.00	C1—C2—C3	121.9 (3)
H2N2—N2—H1N2	120.00	C1—C2—C6	120.1 (3)
C8—N4—H1N4	120.00	C3—C2—C6	117.9 (2)
H2N4—N4—H1N4	120.00	C2—C3—C4	121.7 (2)
C8—N4—H2N4	120.00	C3—C4—C5	119.42 (19)
C10—N5—H1N5	120.00	S1—C5—C4	120.19 (15)
C8—N5—H1N5	120.00	S1—C5—C7	120.05 (15)
N3—C8—N4	121.04 (16)	C4—C5—C7	119.71 (18)
N4—C8—N5	117.84 (16)	C2—C6—C7	121.5 (2)
N3—C8—N5	121.13 (16)	C5—C7—C6	119.7 (2)
N1—C9—N2	115.97 (16)	C2—C1—H1A	110.00
N1—C9—N3	125.41 (16)	C2—C1—H1B	109.00
N2—C9—N3	118.62 (15)	C2—C1—H1C	109.00
N1—C10—N5	122.18 (15)	H1A—C1—H1B	109.00
N5—C10—C11	118.46 (16)	H1A—C1—H1C	109.00
N1—C10—C11	119.35 (16)	H1B—C1—H1C	109.00
C12—C11—C16	118.8 (2)	C2—C3—H3	119.00
C10—C11—C12	118.80 (19)	C4—C3—H3	119.00
C10—C11—C16	122.43 (19)	C3—C4—H4	120.00
C11—C12—C13	120.2 (2)	C5—C4—H4	120.00
C12—C13—C14	121.2 (3)	C2—C6—H6	119.00
C13—C14—C15	119.1 (3)	C7—C6—H6	119.00
C14—C15—C16	120.6 (2)	C5—C7—H7	120.00
C11—C16—C15	120.2 (2)	C6—C7—H7	120.00
			
O1—S1—C5—C4	2.32 (18)	N1—C10—C11—C16	−156.7 (2)
O2—S1—C5—C4	−118.00 (16)	N5—C10—C11—C12	−155.5 (2)
O3—S1—C5—C4	123.67 (16)	C16—C11—C12—C13	−0.1 (4)
O1—S1—C5—C7	179.78 (16)	C10—C11—C16—C15	180.0 (2)
O2—S1—C5—C7	59.47 (17)	C10—C11—C12—C13	179.6 (3)
O3—S1—C5—C7	−58.87 (18)	C12—C11—C16—C15	−0.3 (4)
C10—N1—C9—N3	2.6 (3)	C11—C12—C13—C14	0.6 (6)
C10—N1—C9—N2	−177.59 (17)	C12—C13—C14—C15	−0.5 (6)
C9—N1—C10—N5	−1.3 (2)	C13—C14—C15—C16	0.0 (5)
C9—N1—C10—C11	179.67 (16)	C14—C15—C16—C11	0.4 (4)
C8—N3—C9—N2	176.08 (17)	C1—C2—C6—C7	175.7 (2)
C8—N3—C9—N1	−4.1 (3)	C1—C2—C3—C4	−177.5 (2)
C9—N3—C8—N5	4.3 (3)	C6—C2—C3—C4	1.0 (4)
C9—N3—C8—N4	−176.10 (17)	C3—C2—C6—C7	−2.9 (4)
C10—N5—C8—N4	177.08 (16)	C2—C3—C4—C5	1.7 (3)
C8—N5—C10—C11	−179.23 (16)	C3—C4—C5—C7	−2.5 (3)
C10—N5—C8—N3	−3.3 (3)	C3—C4—C5—S1	174.99 (16)
C8—N5—C10—N1	1.7 (3)	S1—C5—C7—C6	−176.79 (17)
N5—C10—C11—C16	24.2 (3)	C4—C5—C7—C6	0.7 (3)
N1—C10—C11—C12	23.6 (3)	C2—C6—C7—C5	2.1 (4)

### Hydrogen-bond geometry (Å, º)

Cg1 and Cg3 are the centroids of the N1/C9/N3/C8/N5/C10 and C2–C5/C6/C7 rings, respectively.

**Table:**

*D*—H···*A*	*D*—H	H···*A*	*D*···*A*	*D*—H···*A*
N4—H2*N*4···O3^i^	0.86	2.10	2.877 (2)	150
N4—H1*N*4···O3	0.86	2.13	2.950 (2)	160
N2—H2*N*2···N3^ii^	0.86	2.25	3.089 (2)	164
N2—H1*N*2···O1^iii^	0.86	2.05	2.895 (2)	169
N5—H1*N*5···O2	0.86	1.95	2.789 (2)	165
C16—H16···O2	0.93	2.40	3.210 (3)	146
S1—O1···*Cg*1^iv^		2.93 (1)	4.1695 (8)	142 (1)
*Cg*3—*Cg*3			3.9192 (13)	

Symmetry codes: (i) −*x*+1, −*y*+2, −*z*+2; (ii) −*x*+2, −*y*+2, −*z*+2; (iii) *x*+1, *y*, *z*; (iv) −*x*+1, −*y*+2, −*z*+1; (v) *x*, −*y*+3/2, *z*+1/2.
